# Research on Pachymaran to Ameliorate CsA-Induced Immunosuppressive Lung Injury by Regulating Microflora Metabolism

**DOI:** 10.3390/microorganisms11092249

**Published:** 2023-09-07

**Authors:** Chun Ye, Zi-Han Gao, Kai-Qin Chen, Fang-Guo Lu, Ke Wei

**Affiliations:** Medicine School, Hunan University of Chinese Medicine, Changsha 410208, China; yechun19@163.com (C.Y.); 17674341532@163.com (Z.-H.G.); ckq981222@163.com (K.-Q.C.); 001196@hnucm.edu.cn (F.-G.L.)

**Keywords:** pachymaran, immunosuppression, lung injury, flora metabolism, cyclosporine A

## Abstract

Pachymaran (PCP), the major medicinal constituent of Poria cocos, has a regulatory effect on immunosuppressive lung injury, but its mechanism of action with respect to gut microorganisms and their metabolites is not clear. The aim of this study was to investigate the protective effect of PCP against immunosuppressive lung injury caused by cyclosporine A (CsA), and to reveal its possible mechanism of action via the comprehensive analysis of 16S rRNA and LC-MS. We demonstrated that PCP was effective at alleviating CsA-induced immunosuppressive lung injury by restoring the organ indices and lung tissue morphology and structure. PCP significantly altered the composition of the gut and lung microbiota in mice with CsA-induced immunosuppressive lung injury by increasing the number of beneficial bacteria from the *Eubacterium nodatum group*, *Eubacterium ventriosum group*, *Akkermansia*, and *Ruminococcus*, and reducing the pathogenic *Rikenellaceae RC9 gut group* to fulfill its immunomodulatory role. In lung tissue microecology, PCP intervention significantly reduced the abundance of *Chryseobacterium*, *Lawsonella*, *Paracoccus*, and *Sediminibacterium* and increased the abundance of *Alloprevotella*. The LC-MS results showed that PCP alleviated the CsA-induced immunosuppression of lung tissue injury. The model serum metabolite Americine decreased the expression of PC(O-18:1(4Z)/0:0). Our results suggest that PCP may be involved in regulating the composition, function, and metabolism of the gut and lung microbiota to reverse CsA-induced immunosuppressive lung injury.

## 1. Introduction

The lung is the most important organ for gas exchange in mammals and represents the largest epithelial surface in direct contact with the external environment. Because of the communication between the respiratory system and the external environment, bacteria, viruses, dust, and other harmful substances from the external environment can easily cause lung infection, especially when the human immune function declines [1]. The immune system function plays an important role in the stable state of the body, and the occurrences of many diseases are related to the immune function. Abnormal immune function has many unpredictable consequences, such as the immune dysfunction of the intestinal mucosa, which can lead to the diarrheal disease of the host [2]. Acute lung injury (ALI) caused by pulmonary infection due to immune system dysfunction has become a major challenge in the clinical diagnosis and treatment of pulmonary infections in China [3]. Acute lung injury is a type of injury to the lung parenchyma, which is a diffuse inflammatory injury in which a variety of inflammatory mediators and immune cells exert a synergistic effect, resulting in a cascading amplification of the inflammatory “waterfall response” [4]. Therefore, it is of great importance to control the excessive inflammatory response and improve the extent of injury to the lung tissue by regulating immunity and secondary infection with pathogenic microorganisms.

Poria cocos is a kind of medicinal and edible mushroom that has both medicinal and edible effects and has been used as medicine in China for more than 2000 years [5]. Pachymaran is the main ingredient of Poria cocos, which has a variety of biological activities, such as immune regulation, anti-tumor [6], anti-inflammation, anti-oxidation, antibacterial [7], and liver protection [8,9]. Poria cocos is a species that has been used in East Asia for centuries as a traditional medicine and functional food. Poria cocos belongs to the genus Polyporaceae, which is one of the “four monarchs and eight treasures” of traditional Chinese medicine. Studies have shown that PCP can regulate immunity by increasing the expressions of immune factors *IL-6*, *TNF-α*, and *IL-2* [10]. However, the molecular mechanisms by which PCP plays an immunomodulatory role have not yet been scientifically explored and need further research and development.

In the human body, the intestine is the largest digestive and immune organ, playing a key role in host immunity. In recent years, people have been thinking about the health of the gut. Many studies have shown that oxidative stress, inflammation, damage to the intestinal barrier, and an imbalance of intestinal flora can lead to a number of intestinal diseases and other problems. Microbial imbalances can lead to autoimmune diseases, inflammatory bowel disease, and asthma [11]. The metabolism of microenvironmental flora has an important influence on the drug composition and immune status [12]. In addition, pulmonary infection also affects the intestinal flora. Studies have indicated that there are many differences in the composition of the intestinal microflora between patients with lung diseases, such as bronchiolitis and pulmonary tuberculosis, and healthy people [13]. Therefore, the systematic analysis of changes in microecological flora and metabolites is helpful to better understand the regulatory effect of drug molecules on immunity and provides an important avenue for the in-depth analysis of the curative effect and mechanism of Poria cocos polysaccharide. In this work, the model of lung tissue injury caused by the immunosuppression by CsA was established, and the mechanism of the improvement in lung tissue injury via PCP was analyzed in depth based on 16S rRNA sequencing and LC-MS research to determine the application value of natural polysaccharide in the prevention and treatment of lung tissue injury.

## 2. Materials and Methods

### 2.1. Establishment of Animal Model

A total of 45 BLAB/c mice (18~22 g, specific pathogen-free (SPF), 23 males and 22 females) were purchased from Hunan Shrek Jingda Experimental Animal (animal production license number SCXK (Xiang 2019-0004)). After 3 days of adaptive feeding, the 45 mice were randomly divided into a control group (n = 15), model group (n = 15), and pachymaran group (PCP) (n = 15). First, the model group and the PCP group received an intraperitoneal injection of CsA (0.2 mL, 45 mg/kg) (Number: HYM000082991) for 7 days to establish a model of immunosuppressive lung injury, and the control group was injected with the same amount of PBS. After 7 days, the PCP group received 100 mg/kg PCP (Hunan Futian Pharmaceutical Co., Changsha, China) (a 0.2 mL gastric perfusion was administered); the control group and the model group received 0.2 mL of PBS as gastric perfusion for 7 days. During the experiment, all the experimental designs were approved by the Ethics Committee of Hunan University of Traditional Chinese Medicine. All methods were performed in accordance with the relevant guidelines and regulations.

### 2.2. Each Organ Index

Seven days after administration, the samples were collected, and the body mass, lung mass, spleen mass, and thymus mass of the mice in each group were weighed, and the spleen index of each group was calculated as follows: spleen index (%) = spleen mass (g)/mouse body mass (g) × 100%. The lung index and thymus index are the same as above.

### 2.3. Analysis of the Hematoxylin–Eosin Staining of the Lungs

Mouse lung tissue was first soaked and fixed with 4% paraformaldehyde for 1 week, embedded in xylene (Cat#10023418, Shanghai, China, Sinopharm Group Chemical Reagent Co., Ltd.), stained with hematoxylin (Cat#517-28-2, MACKLIN) for 5 min, stained with eosin (Cat#15086-94-9, MACKLIN) for 3 min, and then the tissue sections were sealed in gradient ethanol (Cat#100092683, Sinopharm Group Chemical Reagent Co., Ltd.) and neutral gum (Cat#10004160, Sinopharm Group Chemical Reagent Co., Ltd.) to observe the histopathological changes of the mouse lung tissue under the microscope.

### 2.4. Detection of Colonic Fecal Matter and Lung Tissue via 16S rRNA Sequencing

The Hipure Stool DNA Kit was used to extract total DNA, PCR from feces, and lung tissue to amplify the V3~V4 variable region of the bacterial 16S rRNA gene. Using the forward primer 341F: 5′-CCTACGGGNGGCWGCAG-3′ and the reverse primer 806R: 5′-GGACTACHVGGGTATCTAAT-3′, the purified amplification product was connected to the sequencing compound, and the sequencing library was constructed. The Illumina HiseqTM 25004000 gamma was sequenced on the computer.

### 2.5. Determination of Serum Metabolites via LC-MS

Mouse serum samples were removed from the refrigerator at −80 °C and thawed at room temperature. Samples were collected on an ACQUITY UPLC HSS T3 column (100 mm × 2.1 mm, 1.8 um). The mass spectrometry signal of the samples was collected and separated into positive- and negative-ion scanning modes. QC was injected at regular intervals throughout the experiment. Metabolites were qualitatively analyzed using The Human Metabolome Database (HMDB), LIPID MAPS, and the METLIN and EMDB2.0 databases.

### 2.6. Enzyme-Linked Immunosorbent Assay (ELISA)

After the mouse serum was directly diluted, the concentrations of *IL-6* and *TNF-α* were detected in the serum and lung tissue according to the instructions of the ELISA kit (Shanghai, China, enzyme-linked biology).

### 2.7. Analysis of mRNA Expression via Quantitative PCR (qPCR)

Lung tissues were collected from the refrigerator at −80 °C, and total RNA was extracted with Trizol (lot number #218715, Beijing, China, Tiangen biochemical Technology Co., Ltd.). cDNA NovoStartRSYBR qPCR SuperMix plus was synthesized using a reverse transcription kit (Jiangsu, China, lot number E096-01A Novoprotein Co., Ltd.). The levels of *IL-1β*, *IL-10*, and *IL-6* mRNA (the applied primer sequence supplements in Table 1) were determined via real-time quantitative PCR reaction (LightCycler96 PCR instrument). GAPDH was used as an internal reference, and 2-_∆∆_ Ct was used to analyze the relative expression of mRNA.

### 2.8. Data Processing

All the experimental data from the mice were expressed as “mean ± standard deviation (x ± s)”. Several groups of measured data were tested for the control distribution and homogeneity of variance, and a one-way analysis of variance was used as a control. If the variance was uniform, then LSD was used for multiple comparison, and if the variance was not uniform, then the Games-Howell test was used for multiple comparison. When the control distribution was not satisfied, the rank sum test was used to compare the differences between groups, and the nonparametric rank sum test for independent samples (Kruskal–Wallis ANOVA) was used for pairwise comparison. Data results were processed using SPSS26.0 software.

## 3. Results

### 3.1. PCP Alleviates Immunosuppressive Lung Injury Caused by CsA

**Figure 1:**
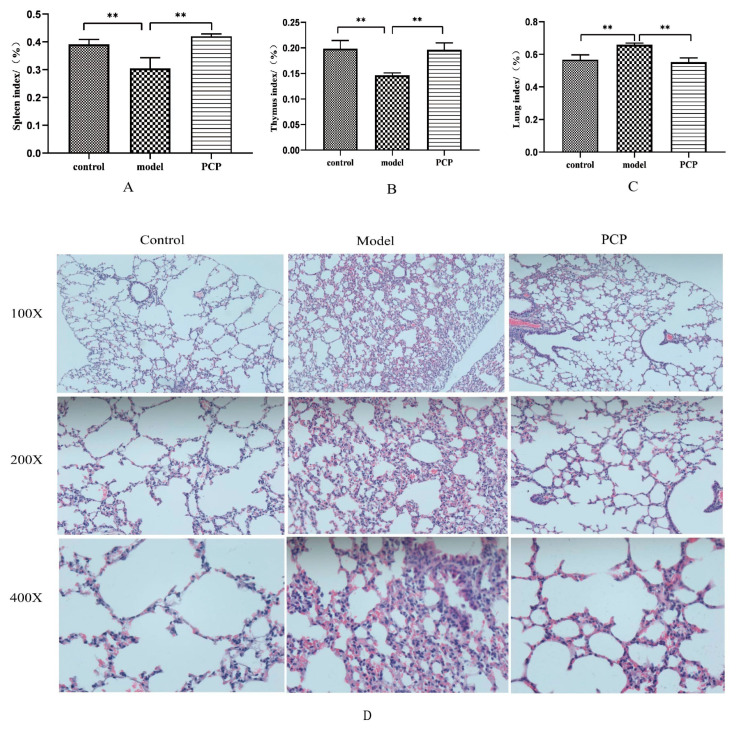
PCP alleviates CsA-induced immunosuppressive lung injury. (**A**) Spleen index of mice in each group (n = 10). (**B**) Thymus index of mice in each group (n = 10). (**C**) Lung index of mice in each group (n = 10). (**D**) HE staining of lung tissue (n = 3). Significant differences are indicated by ** *p* < 0.01.

Compared to the control group, the mice in the model group experienced significant weight loss, and both the thymus and spleen indices decreased, indicating the success of our immunosuppressive CsA model (Figure 1A,B, Table 2). Compared with the model group, the thymus and spleen indices of PCP were significantly increased after 7 days of administration. The results showed that the immunity of PCP was restored to some extent in the mice. Compared with the control group, the lung index of the model group increased significantly, indicating that the immunosuppressive model by CsA affected the injury of lung tissue. After treatment with PCP, the lung index recovered (Figure 1C). To better understand the morphological changes in the lung tissue, we used HE staining to observe the morphological changes in the lung caused by CsA-induced immunosuppression. As shown in Figure 1D, the morphology of the alveoli in the control group was intact and there was no infiltration of inflammatory cells in the lungs, while hyperemia and edema, alveolar collapse, and a large number of immune cell infiltrations in the model group indicated pathological damage to the lung tissue. It is worth noting that the degree of lung tissue injury significantly improved in the PCP-treated mice compared with the model group, hyperemia and edema decreased, the alveolar wall thickened, the infiltration of inflammatory cells decreased, and the necrotic consolidation area decreased. Therefore, we can conclude that PCP can preserve the morphology and structure of lung tissue.

### 3.2. PCP Regulates the Microecological Diversity and Composition of Lung Tissue

**Figure 2:**
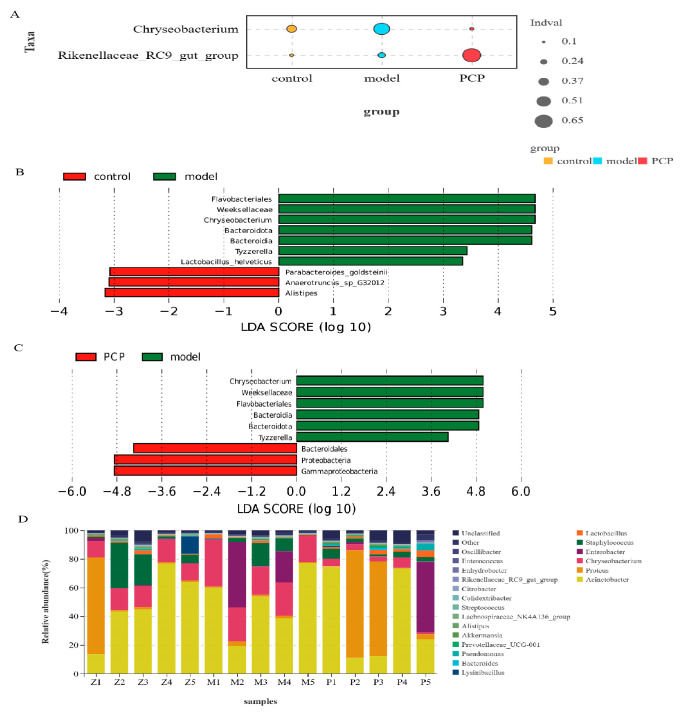
PCP regulates the microecological diversity and composition of lung tissue. Note: Z: control group (n = 5); M: model group (n = 5); P/PCP: PCP group (n = 5). (**A**) Indicator analysis (indicator analysis calculates the indicator value (IndVal) of each species in each grouping based on the abundance and frequency of the occurrence of the species in the sample. The higher the value, the more likely the species is to be the indicator species of the grouping). (**B**,**C**) LEfSe analysis (by analyzing the differences in microbiota between groups using LEFse, it is possible to identify the main flora specific to each group (≥2 groups), which is useful for biomarker development and other studies). (**D**) Species distribution map of microbial community at genus level (lung microecology).

Based on the lung flora results, we found that their dilution curves tended to be smooth, indicating that the amount of sequencing data from the samples was sufficient. The effective sequence of each sample of lung flora was more than 50,000, indicating that the flora was effectively detected, and the coverage index (good coverage) of each sample was higher than 0.998, indicating that the sequencing covered most of the microflora in each lung tissue sample. The results in supplementary Table 3 show that the *α*-diversity indices of each group (Shannon index, Simpson index, Chao index, and ACE index) were not statistically significant, indicating that there was no significant difference in the number of individual species in the lung flora of each group (i.e., the species richness and uniformity of the lung flora of each group tended to be the same, but the species structure and composition of the lung flora of each group and the comparison between groups still needed *β*-diversity). In this study, PLS-DA was used to analyze the distribution characteristics of the lung flora in the lung tissue samples of the mice in each group and was combined with Adonis analysis to determine the significant differences. It was found that T1 and T2 explained 30.9% and 19.5% of the data changes in the lung structure of the mice, respectively (Figure S1A). The results of the indicator analysis showed that there were significant differences in the structure of the lung flora between the control, model, and PCP groups (Figure 2A). We also analyzed the differences in the microflora between groups using LEfSe (LDA > 3), which was used to analyze the results of all classification levels (Figure 2B,C and Figure S1B,C). Compared with the control group, the model group had mainly *Flavobacteriales*, *Weeksellaceae*, and *Chryseobacterium* as indicator species. After treatment with PCP, the indicator species increased significantly, such as *Proteobacteria*, *Gammaproteobacteria*, *Bacteroidales*, etc.

To further compare the differences in the species composition, we examined the 20 most abundant species at the phylum and genus levels, and we used heat maps to distinguish the microbial community at the phylum (Figure S2A) and genus (Figure 2D) levels. Compared to the control group, the abundance of *Bacteroidota* (*p* = 0.015) significantly increased in the model group, while the abundance of *Planctomycetota* (*p* = 0.011) significantly decreased. After PCP treatment, mainly *Proteobacteria* (*p* = 0.03) were upregulated and *Bacteroidota* (*p* = 0.008) and other species were decreased. At the genus level, the abundance of *Chryseobacterium* was significantly increased in the model group compared with the control group; after the PCP intervention, the abundance of *Chryseobacterium* decreased significantly. After treatment with PCP, the abundance of *Lawsonella*, *Paracoccus*, and *Sediminibacterium* decreased, while the abundance of *Alloprevotella* increased. The results indicate that PCP can regulate the relative abundance of the lung microecology.

### 3.3. PCP Regulates the Diversity and Composition of the Gut Microbiota

**Figure 3:**
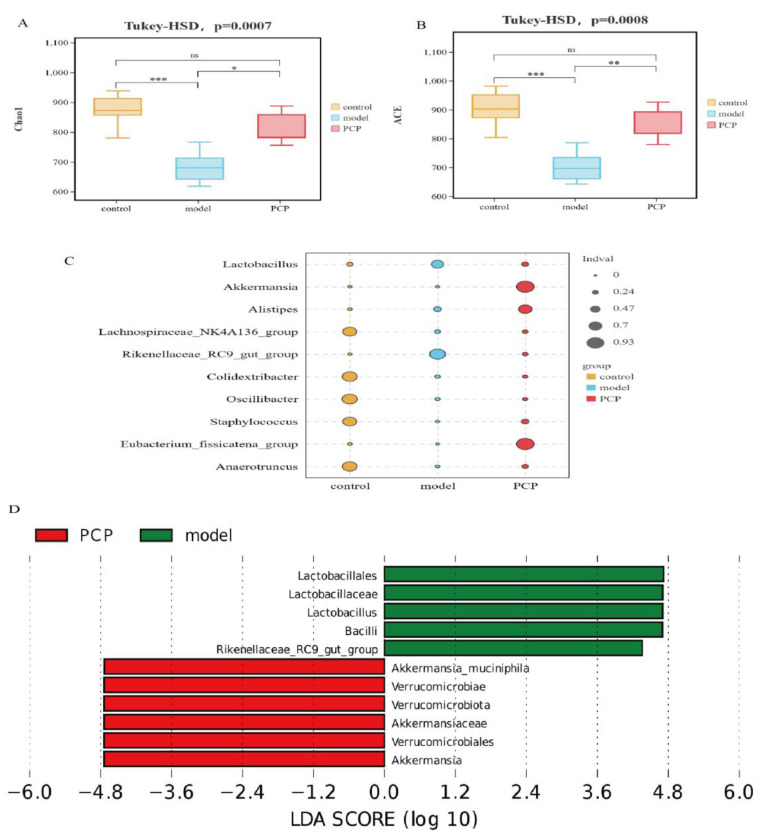
PCP regulates the diversity and composition of the gut microbiota. Note: Z: control group (n = 5); M: model group (n = 5); P/PCP: PCP group (n = 5). (**A**) Chao index (gut microecology). (**B**) ACE index (gut microecology). (**C**) Indicator analysis (gut microecology) in genus, *p* < 0.05. (**D**) LEfSe analysis (gut microecology), LDA > 4. Significant differences are indicated by * *p* < 0.05, ** *p* < 0.01, and *** *p* < 0.001.

Based on the gut flora results, the effective sequence of each sample was more than 80,000 and the Good’s coverage of each sample was higher than 0.997. We used the Chao index and ACE index to evaluate the species richness of the samples. The results show (Figure 3A,B) that the Chao index and ACE index of the model group were significantly lower than those of the corresponding control group. Importantly, PCP intervention can restore the species richness of the microbial community. However, there was no significant difference between the Shannon index and Simpson index. Through PLS-DA analysis of the *β*-diversity and Adonis test results, it was found that there were significant changes in the gut flora in each group. Compared with the control, the structure of the intestinal flora in the model mice changed significantly (*p* = 0.011) and could be improved via PCP intervention (Adonis, Bray–Curtis metric, *p* = 0.007, R^2^ = 0.1793), indicating that PCP could regulate the structure of intestinal flora (Figure S3A). The results of the indicator analysis showed that there were significant differences in the structure of the intestinal flora between the control, model, and PCP groups (Figure 3C). We analyzed the differences in the flora between the groups using LEfSe (LDA > 4, linear discriminant analysis) to analyze the results of all classification levels (Figure 3D and Figure S2B–D). Compared with the control group, the model group mainly used *Lactobacillus*, *Bacteroidota*, *Bacteroidales*, and the *Rikenellaceae_RC9_gut_group* as the main indicators. After PCP intervention, the main indicators were *Verrucomicrobiota*, *Verrucomicrobiae*, *Akkermansia,* etc.

**Figure 4:**
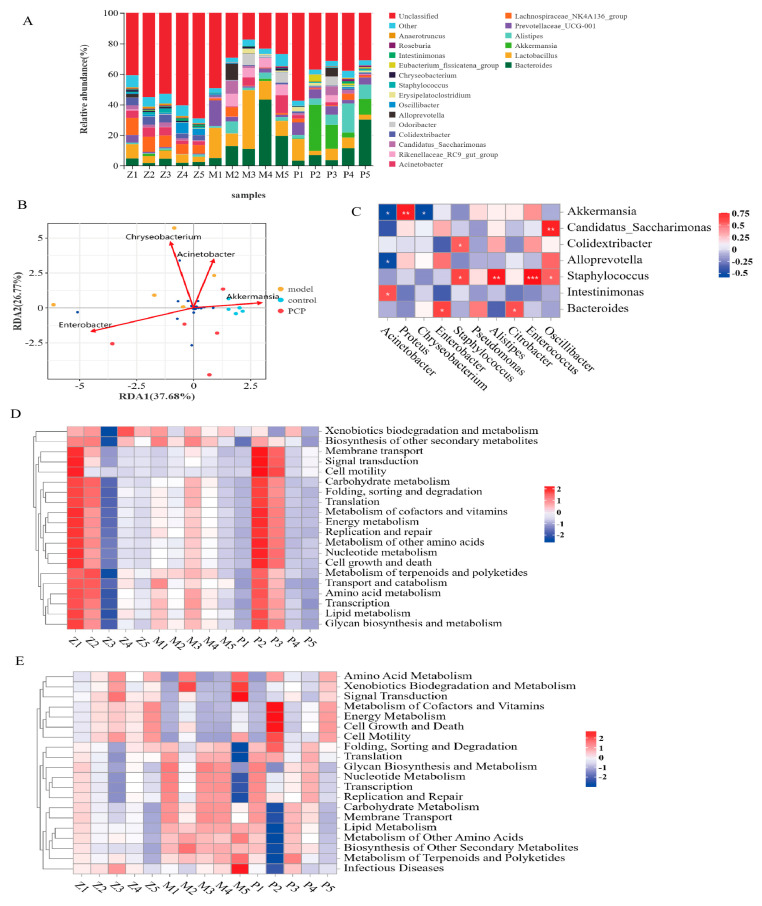
PCP alleviates the changes in intestinal microflora in immunosuppressive lung injury induced by CsA (note: Z: control group (n = 5); M: model group (n = 5); P/PCP: PCP group (n = 5). (**A**) Species distribution map of microbial community at genus level. (**B**,**C**) Correlation analysis of gut flora and lung flora. (**D**) Prediction analysis of PICRUSt2 function of lung flora (PICRUSt2 function based on species annotation and abundance information from OTU, functional annotation of the KEGG pathway using the Integrated Microbial Genomes database(IMG) bacteria/archaea (16S), or fungal (ITS) MetaCyc Pathway prediction, and the counting of the abundance information for each pathway). (**E**) Prediction analysis of Tax4Fun function of gut flora (Tax4Fun first associated the prokaryotic 16S rRNA sequences of existing genomes in the KEGG database with the 16S rRNA sequences in the SILVA database, and then interrupted the sequences of the existing prokaryotic species genomes in the KEGG database and used UProC to count the KO sequences of all genomes). Finally, the copy number of 16S was used to correct the number of species, and KEGG prediction and KO abundance statistics were implemented. Significant differences are indicated by * *p* < 0.05, ** *p* < 0.01. *** *p* < 0.001.

We selected the 20 species with the highest abundance at the phylum and genus levels, and we used heat maps to reveal significant differences in the bacterial taxonomic maps between the phylum (Figure S3B) and genus (Figure 4A) levels. Compared to the control group, the abundance of *Bacteroidota* (*p* = 0.03) at the phylum level significantly increased in the model group, and the abundance of *Verrucomicrobiota* (*p* = 0.015) significantly increased after PCP treatment. At the genus level (Figure S3C,D), the abundance of the model group was significantly increased in *Lactobacillus* (*p* = 0.015), *Bacteroides* (*p* = 0.008), the *Rikenellaceae RC9 gut group* (*p* = 0.015), and *Alistipes* (*p* = 0.008), compared with the control group. However, the abundance decreased significantly in *Colidextribacter* (*p* = 0.008), the *Lachnospiraceae_NK4A136_group* (*p* = 0.015), the *Eubacterium_nodatum_group* (*p* = 0.03), and *Oscillibacter* (*p* = 0.03). PCP could significantly downregulate the abundance of the *Rikenellaceae RC9 gut group* (*p* = 0.015); it could also increase the abundance of the *Eubacterium_fissicatena_group* (*p* = 0.008), *Eubacterium_xylanophilum_group* (*p* = 0.008), *Akkermansia* (*p* = 0.015), and *Ruminococcus* (*p* = 0.03). Thus, these results suggest that PCP can significantly regulate the relative abundance of intestinal flora.

### 3.4. Analysis of the Relationship between Intestinal Flora and Lung Flora

We analyzed the relationship between the first 18 species at the genus level with the highest richness in lung microecology and gut microecology (Figure 4B,C). We found that the most closely related lung microecology species were *Acinetobacter*, *Chryseobacterium*, and *Enterobacter*. Among them, *Chryseobacterium* was positively correlated with *Lactobacillus* and the *Rikenellaceae_RC9_gut_group* in the gut microecology, and negatively correlated with *Akkermansia*, *Staphylococcus*, and the *Eubacterium_fissicatena_group* in the gut microecology. There was a positive correlation between *Acinetobacter* and *Intestinimonas*, a negative correlation between *Enterobacter* and *Prevotellaceae_UCG-001* (*Prevotellaceae UCG-001* is a beneficial bacterium that has an anti-inflammatory effect and can alleviate the disorder of glucose and lipid metabolism), and a positive correlation with *Roseburia*.

### 3.5. Prediction and Analysis of Metabolic Function of Pulmonary Microflora

Based on the results of the differential species analysis, the function prediction analysis of PICRUSt2 was performed, as shown in Figure 4D. The function analysis of six kinds of biological metabolic pathways, including metabolism, genetic information processing, environmental information processing, cellular processes, organismal systems, and human diseases, was carried out. Among them, the change in organismal systems was the most significant. Compared with the control group, the predictive gene abundance of the metabolic pathway of the digestive system (digestive system, *p* = 0.015) was significantly upregulated in the model group, and the organismal systems (digestive system, *p* = 0.008; endocrine system, *p* = 0.03) could be significantly downregulated after the PCP intervention.

### 3.6. Prediction and Analysis of the Metabolic Function of the Intestinal Flora

Based on the results of the differential species analysis, the function prediction analysis of Tax4Fun was performed, as shown in Figure 4E, including metabolism, genetic information processing, environmental information processing, cellular processes, organismal systems, and human diseases. Compared with the control group, the predicted gene abundance of intestinal flora in the model group was higher in the metabolic pathways of metabolism (carbohydrate metabolism, lipid metabolism, metabolism of other amino acids, metabolism of terpenoids and polyketides, biosynthesis of other secondary metabolites), organismal systems (endocrine system, digestive system), human diseases (neurodegenerative diseases, immune diseases), environmental information processing (membrane transport), cellular processes (cell communication), and 11 secondary functional layers. After PCP intervention, the predictive gene abundance was significantly downregulated (*p* < 0.05) in the metabolic pathways of three primary functional layers and five secondary functional layers of lipid metabolism, the metabolism of other amino acids, the biosynthesis of other secondary metabolites, and the digestive system and signaling (molecules and interaction).

### 3.7. Multivariate Statistical Analysis of the Distribution of Serum Metabolites and the Identification of Their Metabolites

**Figure 5:**
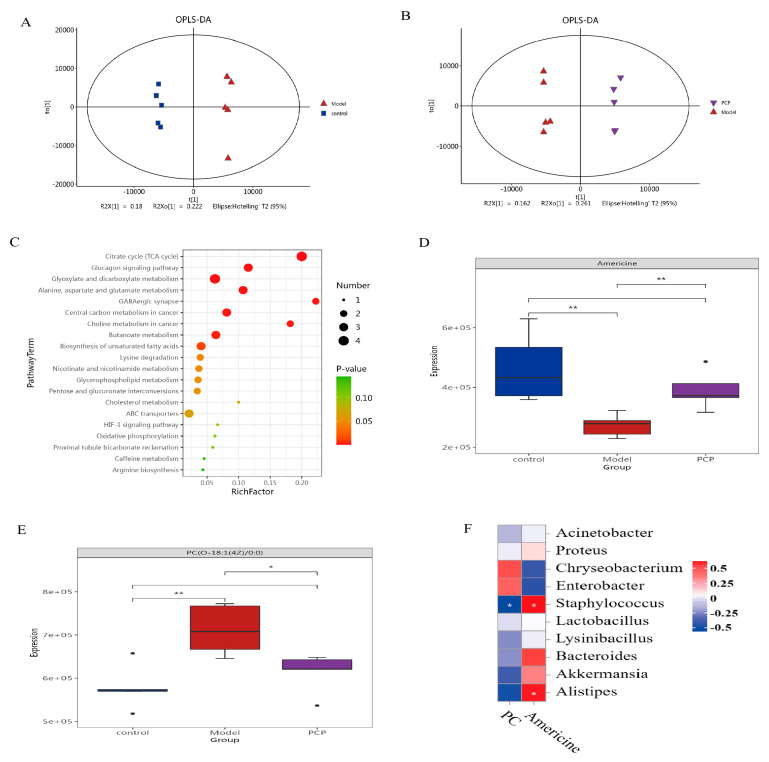
Multivariate statistical analysis of the distribution of serum metabolites and the identification of their metabolites. Note: control group (n = 5); model group (n = 5); PCP group (n = 5). (**A**,**B**) Orthogonal partial least-squares analysis (OPLS-DA) was used to distinguish overall differences in metabolic profiles between groups and to find different metabolites between groups. (**C**) Control vs. model: bubble chart for metabolic pathway enrichment (the ordinate is the name of the metabolic pathway; the abscissa is the enrichment factor (rich factor = number of significantly different metabolites/total number of metabolites in the metabolic pathway; the larger the rich factor, the greater the degree of enrichment; the color from green to red indicates that the *p*-value decreases again; the larger the dot, the greater the number of metabolites enriched in the metabolic pathway). (**D**) Americine. (**E**) PC(O-18:1(4Z)/0:0). (**F**) Correlation analysis of lung flora and metabolism. Significant differences are indicated by * *p* < 0.05, ** *p* < 0.01.

To evaluate the serum metabolite changes in CsA-induced immunosuppressive lung tissue injury, serum samples were analyzed via LC-MS. A total of 2235 metabolites in positive mode and 2678 metabolites in negative mode were recorded and selected for further analysis. As shown in the OPLS-DA diagram (Figure 5A,B), the metabolites of the model group and the control group and the PCP group and the model group were basically separated, indicating that there were significant differences in the metabolism between the groups. A total of 4912 metabolites in serum were detected via LC-MS, and the differential metabolites were analyzed via a VIP > 1 *t*-test of a *p*-value < 0.05 screening. Compared with the control group, 197 differentially expressed metabolites (DEMs) were identified by the model. Compared with the model group, the PCP group identified 178 significantly altered DEMs (Figure 5C and Figure S4), including 78 lipids and lipid-like molecules, 19 organoheterocyclic compounds, 19 organic oxygen compounds, 16 organic acids and derivatives, 10 benzenoids, and other metabolites. Among these peaks, 83 metabolites were upregulated and 114 were downregulated in the model group compared with the control group; compared with the model group, 82 metabolites were upregulated and 96 were downregulated in the PCP group. The analysis of the KEGG metabolic pathways between the PCP and the model group showed that they included ABC transporters, choline metabolism in cancer, amyotrophic lateral sclerosis, D-amino acid metabolism, arginine biosynthesis, purine metabolism, and others.

As mentioned above, when comparing the differences in serum metabolites between the PCP and model groups, a total of 178 metabolites changed significantly. Considering the diversity of their levels in serum, we classified these metabolites according to their relative abundance to measure the close correlation between significant DEMs. Assuming that the correlation between the control, model, and PCP groups was significant at the same time, 20 species of the most relevant DEMs (Figure S5A,B) of the LC-MS were selected as the subject of further investigation. Compared with the control group, mainly 14 species of DEMs were upregulated in the model group, such as phosphatidyl ethanolamine (PE), phosphatidylserine (PS), phosphatidylcholine (PC), lysophosphatidylcholine (Lyso), and bacterialurea diglucoside (for example, PC(O-18:1(4Z)/0:0), PS(18:1(9Z)/21:0), PE(19:0/20:2(11Z,14Z)), LysoPC(20:4(8Z,11Z,14Z,17Z)/0:0), Bacterioruberin, and Diglucoside). PCP could upregulate Americine (Figure 5D) after the intervention and downregulate PC(O-18:1(4Z)/0:0) (Figure 5E). Compared with the model group, some metabolites, such as PC and Lyso, showed a downward trend under the action of PCP, such as lysoPI(20:4(5Z,8Z,11Z,14Z)/0:0), PC(O-18:1(4Z)/0:0), PC(19:0/0:0), and PE-NMe2(15:0/20:2(11Z,14Z)). These results suggest that PCP can ameliorate CsA-induced immunosuppressive injury to lung tissue by regulating metabolite changes.

### 3.8. Correlation Analysis of Microbiome and Metabolic Group

**Figure 6:**
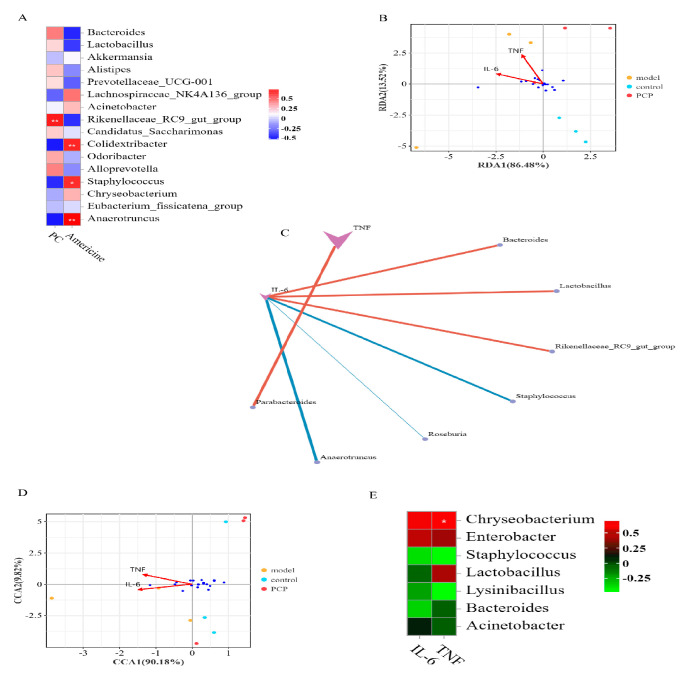
Analysis of the relationship between intestinal and pulmonary flora and cytokines. Note: TNF: *TNF-α*. (**A**) Correlation analysis of lung flora and metabolism. (**B**) RDA analysis (the values in the axis bracket indicate the axis as a percentage of the total change in species diversity) of gut flora. (**C**) Correlation analysis between gut flora and cytokines (A line indicates that there is a correlation, *p* < 0.05). (**D**) CCA analysis (the values in the axis bracket indicate the axis as a percentage of the total change in species diversity) of lung flora. (**E**) Correlation analysis between lung flora and cytokines. Significant differences are indicated by * *p* < 0.05, ** *p* < 0.01.

To further investigate the correlation between the microflora and differential metabolites in serum, a Spearman correlation analysis was performed. We analyzed the correlation between two significant differential metabolites and the flora of the first 20 genera. In the lung flora (Figure 5F), the metabolite PC(O-18:1(4Z)/0:0) was negatively correlated with *Staphylococcus*, whereas Americine was positively correlated with *Staphylococcus* and *Anaerotruncus*. In the gut flora (Figure 6A), the metabolite PC(O-18:1(4Z)/0:0) was positively correlated with the *Rikenellaceae_RC9_gut_group*, and the metabolite Americine was positively correlated with *Anaerotruncus*, *Colidextribacter*, and *Staphylococcus*. Therefore, these metabolites and microorganisms may affect the immune system of the body.

### 3.9. Analysis of the Relationship between Gut and Lung Flora and Cytokines

Based on a redundancy analysis (RDA) and Spearman correlation coefficient analysis, the correlation between “sample-intestinal flora-cytokines” in each group was analyzed. The results showed the following (Figure 6B): at the level of phylum and genus classification, all samples were obviously clustered in one group on the RDA1 (Genus: RDA1 = 86.48%), which was consistent with the respective grouping, indicating that the effects of the intestinal flora and cytokines *IL-6* and *TNF-α* on the sample distribution were consistent with the grouping effect. As shown in Figure 6C, the results of the correlation analysis between the gut flora and cytokines showed that, at the genus level, *Bacteroides*, *Lactobacillus*, and the *Rikenellaceae_RC9_gut_group* were positively correlated with *IL-6*, *Staphylococcus*, *Roseburia,* and *Anaerotruncus* were negatively correlated with *IL-6*, and *Parabacteroides* was positively correlated with *TNF-α*. In the lung flora (Figure 6D,E), based on a canonical correspondence analysis (CCA), there was a positive correlation between *Chryseobacterium* and *TNF-α* in CCA1 = 90.18% at the genus level. It could be seen that this intestinal flora and lung flora jointly influence the secretion and expression of immune-related cytokines.

### 3.10. Effect of PCP on Cytokine Levels and Gene Expression in CsA-Treated Mice

**Figure 7:**
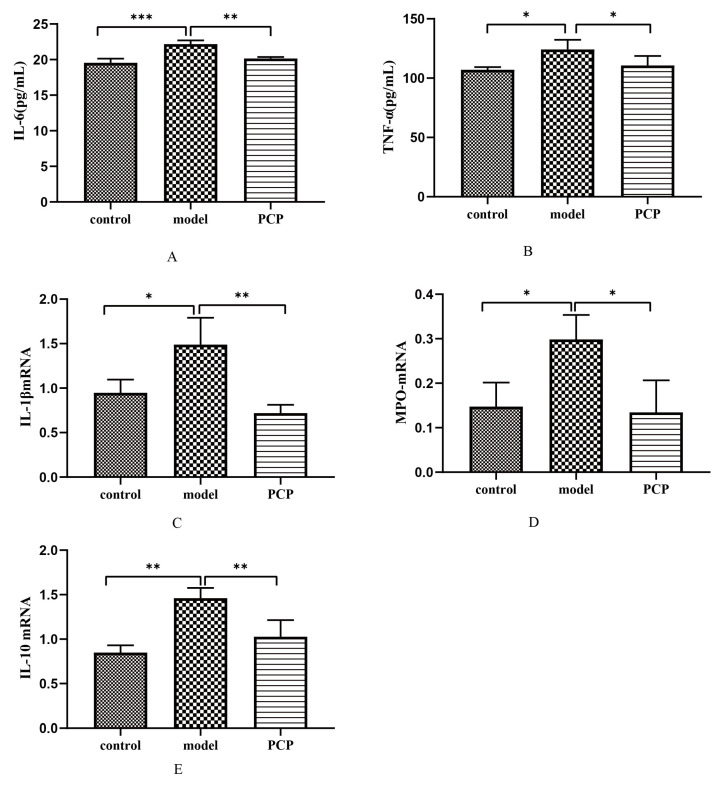
Effects of PCP on cytokines and mRNA expression in CsA-treated mice. (**A**) ELISA detecting serum level of *IL-1β* (n = 3). (**B**) ELISA detecting serum level of *TNF-α* (n = 3). (**C**–**E**) Gene expression levels of *IL-1β*, *MPO*, and *IL-10* at the transcriptional level (n = 3). Significant differences are indicated by * *p* < 0.05, ** *p* < 0.01, and *** *p* < 0.001.

To further investigate the protective effect of PCP on CsA-induced immunosuppressive lung injury, the serum expression levels of pro-inflammatory cytokines *IL-6* and *TNF-α* were determined. The serum results showed that *IL-6* and *TNF-α* significantly increased after CsA infection (*p* < 0.01) (Figure 7A,B). After treatment with PCP, the expressions of *IL-6* and *TNF-α* in serum were significantly decreased. Therefore, PCP is thought to have a protective effect on CsA-induced immunosuppressive lung injury induced by reducing lung inflammation. The levels of *IL-1β*, *MPO*, and *IL-10* mRNA detected via qRT-PCR (Table 1) showed that the levels of *IL-1β* and *MPO* mRNA were significantly increased in the serum of the CsA-infected mice (*p* < 0.01) (Figure 7C,D), while the level of *IL-10* mRNA was significantly decreased (Figure 7E). After PCP treatment, the levels of *IL-1β* and *MPO* mRNA were significantly decreased, and the expression of *IL-10* mRNA was promoted. Therefore, these results suggest that PCP has a protective effect on CsA-induced immunosuppressive ALI.

## 4. Discussion

The immune organ is the main site where the body performs immune functions. As the central immune organ, the thymus is the site where immune cells originate, differentiate, and mature; the spleen is an important peripheral immune organ of the body and a site where mature T and B cells settle [14]. In this study, the spleen index and thymus index of the mice were calculated, and it was found that the indices decreased significantly after CsA modeling, indicating that the immunocompromised mouse model was successfully established. Notably, the CsA-induced immunosuppressive lung injury was pronounced, and the extent of the lung tissue damage was significantly improved after treatment with PCP. This work demonstrated that PCP can ameliorate CsA-induced immunosuppressive lung injury. The intestinal microflora, as the largest and most complex microecosystem in the human body, can regulate the function of the immune system while maintaining the balance of the intestinal microecology. Weak immune function can lead to an imbalance of intestinal flora, which, in turn, leads to an imbalance of the host immune system, which, in turn, promotes the occurrence and development of various diseases. The intestinal flora is very important for the immune response of the intestinal and respiratory tracts and the stability of the internal environment [15]. It has already been shown that a disturbed intestinal microflora can lead to an imbalance of the immune system and aggravate pneumonia [16]. Thus, intestinal flora has become a new target for the treatment of respiratory diseases. Studies have shown that natural polysaccharides can boost immunity by promoting beneficial microorganisms and improving immune cells [17]. We hypothesize that the effect of PCP on lung injury caused by immunosuppression is related to the changes in the intestinal microflora. Therefore, in this study, we extracted the 16S rRNA sequence of bacterial DNA from the contents of mouse ceca to study the intestinal microflora of mice. As expected, the microflora diversity of the mice decreased after CsA injection, as evidenced by the decreases in the Chao index and Shannon index. The results showed that PCP intervention at the phylum classification level was able to restore the species richness of the microbial community and the structure of the intestinal flora. Compared with the model group, *Verrucomicrobiota*, *Verrucomicrobiaeand*, and *Akkermansia* were mainly present after PCP intervention. *Verrucomicrobiota* could produce SCFAs, such as propionic acid and butyric acid, which have protective effects on gut health and immune system regulation [17,18]. At the genus classification level, PCP could significantly decrease the abundance of the *Rikenellaceae RC9 gut group* and increase the abundance of the *Eubacterium_fissicatena_group*, *Eubacterium_xylanophilum_group*, *Akkermansia*, and *Ruminococcus*. Compared with the model group, the Tax4Fun function prediction results showed that the predictive gene abundance of lipid metabolism, the metabolism of other amino acids, the biosynthesis of other secondary metabolites, the digestive system and signaling pathways, and other related pathways decreased after PCP intervention. These results suggest that PCP could play its immunomodulatory role by increasing the beneficial bacteria of the *Eubacterium nodatum group*, *Eubacterium ventriosum group*, *Akkermansia,* and *Ruminococcus* and reducing the pathogenic bacteria of the *Rikenellaceae RC9 gut group* well. It is reported that *Akkermansia* can improve the integrity of intestinal epithelial cells and the thickness of the mucus layer, promote the intestinal health of the host [19], and play an important role in maintaining the balance of the intestinal environment and inhibiting inflammation [20].

In many lung diseases, the ecological composition and changes in lung flora are closely related to lung injury. Lung lesions lead to reduced ventilation, reduced oxygen content, and an accumulation of lactic acid in the alveoli, which leads to a proliferation of bacteria that can utilize lactic acid (e.g., *proteobacteria*). The result of this massive multiplication is further inflammation and the worsening of the lesions in the lung tissue. At the same time, bacteria such as *Lactobacillus* can break down a large amount of lactic acid, which is negatively correlated with the degree of lung damage [21]. Studies have shown that ALI can lead to the disruption of lung microbial flora [22]. In this study, the microecology of normal lung tissue consisted mainly of *Proteobacteria* and *Firmicutes*. At the genus level, the model group could upregulate the abundance of *Chryseobacterium*. Studies have shown that weak immunity or lung infection in patients can easily lead to an increase in *Chrysobacteria* [23]. After treatment with PCP, the abundance of *Chryseobacterium*, *Lawsonella*, *Paracoccus*, and *Sediminibacterium* was significantly decreased, while the abundance of *Alloprevotella* was increased. *Lawsonella* is an opportunistic pathogen and infection can be accelerated when the organism is immunocompromised [24]. *Alloprevotella* produces short-chain fatty acids that maintain the intestinal barrier function and have anti-inflammatory effects [25]. Compared to the microorganisms in the intestine, the number and diversity of microflora in the lungs are lower. The alteration of lung flora is not as obvious as that of intestinal flora, so there is no significant difference in the metabolic function of lung flora, but PCP also partially ameliorates CsA-induced immunosuppressive lung injury. Analyses of the microbial association between the lung and gut revealed that the lung *Acinetobacter*, *Chryseobacterium*, and *Enterobacter* flora were closely related to the changes in the gut flora, and that SCFAs produced by the gut microbiota could be transported to the lung along the gut–lung axis to modulate pulmonary immunity [26]. Therefore, we speculate that the microbiota of the pulmonary gut flora may interact with each other to influence the immune system.

Studies have shown that organic acids can enhance the host immune response and regulate the host expression of inflammatory factors, including *IL-6*, *IL-10*, *IL-1β*, etc. [27]. The results of the LC-MS showed that Americine, as an organic acid and its derivative, showed a downward trend under the condition of immunosuppression, and PCP intervention could promote the production of this metabolite and improve host immunity. As a part of the cell membrane, the metabolic disorder of glycerol phospholipid is the core of inflammation-induced lung injury [18,28]. The results showed that the proportion of glycerol phospholipid metabolites was the largest and most affected in the lung injury model of the CsA-induced immunosuppressive mice. Thus, lysoPC(22:6(4Z,7Z,10Z,13Z,16Z,19Z)/0:0), lysoPC(20:4(8Z,11Z,14Z,17Z)/0:0), and lysoPC(20:5(5Z,8Z,11Z,14Z,17Z)/0:0) were significantly upregulated in the model group. It has been suggested that glycerol phospholipid metabolites may further promote inflammation in ALI by altering lipid metabolism, and the activation of lyso may be involved in the inflammatory process [29]. PCP intervention can partially modulate LysoPC(0:0/18:2(9Z,12Z)), LysoPE(20:5 (5Z,8Z,11Z,14Z,17Z)/0:0), and PC(O-18:1(4Z)/0:0), which, in turn, alleviates the perturbation of glycerophospholipid lipid metabolism. In our study, we found that 78 different lipids changed after PCP intervention, accounting for 44% of the total different metabolites. Therefore, we hypothesize that PCP may inhibit CsA-induced immunosuppressive lung injury by regulating the production of lipids and lipid-like molecular metabolites, such as glycerol phosphate metabolites. In the differential metabolism we discovered, PCP significantly ameliorated the significant changes in the metabolites Americine and PC(O-18:1(4Z)/0:0) in the serum of mice with immunosuppressive lung tissue injury induced by CsA. However, these metabolites do not have all KEGG pathway information, suggesting that they are not included in the database. Based on the above, we speculate that the targeting of gut microflora and associated metabolites by PCP may be used in CsA-induced immunosuppressive lung injury. According to the association between the changes in flora and these two types of metabolites, we found that these metabolites were positively/negatively correlated with *Rikenellaceae_RC9_gut_group*, *Staphylococcus*, *Anaerotruncus*, and *Colidextribacter*. There may be some relationship between these microorganisms and glycerol phospholipids. If we know the pathway of metabolites, then we can explore the close relationship between metabolite-related mechanisms and microbial communities.

The results of the correlation analysis between intestinal flora and cytokines showed that the content of the pro-inflammatory factor *IL-6* was positively correlated with the abundance of *Bacteroides*, *Lactobacillus*, and *Rikenellaceae_RC9_gut_group*, whereas *Staphylococcus*, *Roseburia*, and *Anaerotruncus* were negatively correlated with the *IL-6* content. There was a positive correlation between *Parabacteroides* and *TNF-α*. After PCP treatment, the level of the beneficial bacteria *Lactobacillus* may be increased. *Lactobacillus* is a type of probiotic symbiotic bacteria in the gastrointestinal tracts of humans and animals that accompany the host life. *Lactobacillus* is reported to restore the imbalance of intestinal microflora by producing pro-inflammatory cytokines and upregulating SCFAs [30]. *Staphylococcus* is also a highly pathogenic bacterium in humans [31]. Thus, PCP can reduce the imbalance of intestinal flora in mice with CsA-induced immunosuppressive lung injury by increasing the diversity of intestinal microflora, altering the relative abundance of some bacteria, and playing a role in inflammation and immune regulation through various pathways of regulation. We believe that part of the mechanism of PCP in the healing and repair of CsA-induced immunosuppressive lung injury is due to the remodeling of the intestinal microbiota.

The imbalance of the inflammatory response is an important cause of ALI, and the regulation of the imbalanced inflammatory response is one of the key mechanisms for the prevention and treatment of ALI [32]. According to related studies, neutrophils usually accumulate at sites of inflammation to produce *MPO*, and the excessive production of oxidants can lead to oxidative tissue damage. In inflammatory tests in animals, the increase in the *MPO* content is often taken as a sign of an inflammatory response. As pro-inflammatory factors, *IL-2*, *IL-6*, and *TNF-α* can stimulate the production of other inflammatory factors and help the body to complete the immune response [33]. In this study, after intervention with PCP, the expressions of *IL-6* and *TNF-α* were significantly inhibited in the serum of mice with CsA-induced lung injury. PCP may also reduce CsA-induced lung injury by decreasing the levels of *IL-1β* and *MPO* mRNA and promoting *IL-10* mRNA. Therefore, PCP can be used as a potential drug for the treatment of CsA-induced immunosuppressive lung injury.

In conclusion, PCP may ameliorate the CsA-induced immunosuppressive injury to lung tissue. The mechanism might be to restore the diversity of microorganisms in the gut and lungs and the changes in serum metabolites to inhibit inflammation. However, in this study, it is not clear how the gut flora and metabolites interact with PCP in restoring CsA-induced immunosuppressive lung injury, which is a direction our team will explore further.

## Figures and Tables

**Table 1:** Primer sequences for qRT-PCR.

Gene	Upstream Primer	Downstream Primer
*IL-1β*	5′-AAAGCTCTCCACCTCAATGG-3′	5′-CCCAAGGCCACAGGTATTT-3′
*MPO*	5′-AGATCCGGGAGCGACTATTT-3′	5′-GCGTCTCCAGGCATTGTATC-3′
*IL-10*	5′-TTGAATTCCCTGGGTGAGAAG-3′	5′-TCCACTGCCTTGCTCTTATTT-3′

**Table 2:** The body weights of the mice (note: Z: control group (n = 15); M: model group (n = 15); P: PCP group (n = 15)).

Group	Weight	Group	Weight	Group	Weight
Z1	23	M1	25	P1	25
Z2	22	M2	22	P2	25
Z3	21	M3	23	P3	24
Z4	24	M4	23	P4	24
Z5	25	M5	22	P5	24
Z6	22	M6	24	P6	25
Z7	24.6	M7	23	P7	25
Z8	22	M8	23	P8	26
Z9	24	M9	24	P9	24
Z10	23	M10	24	P10	25
Z11	24	M11	22	P11	26
Z12	22	M12	24	P12	24
Z13	24	M13	23	P13	24
Z14	24	M14	21	P14	23
Z15	25	M14	22	P15	24

**Table 3:** Lung microecology: alpha diversity index (x ± S, n = 5).

Group	Shannon	Simpson	Chao	ACE
Control	2.40 ± 0.78	0.62 ± 0.16	415.40 ± 52.30	434.39 ± 61.49
Model	2.28 ± 0.40	0.68 ± 0.08	395.63 ± 69.93	414.80 ± 76.38
PCP	2.56 ± 0.60	0.58 ± 0.14	500.82 ± 158.90	515.91 ± 168.13

Note: Compared with the control group, the model group and the PCP group in Chao, Shannon, Simpson, and ACE correlation significance: *p* > 0.05. Compared with the model group, the PCP group in Chao, Shannon, Simpson, and ACE correlation significance: *p* > 0.05.

## Data Availability

Not applicable.

## Supplementary Materials

The following supporting information can be downloaded at: https://www.mdpi.com/article/10.3390/microorganisms11092249/s1.

## References

[1] Kumar V. (2020). Pulmonary Innate Immune Response Determines the Outcome of Inflammation During Pneumonia and Sepsis-Associated Acute Lung Injury. Front. Immunol..

[2] Ding S., Jiang H., Fang J. (2018). Regulation of Immune Function by Polyphenols. J. Immunol. Res..

[3] Liu Y., Xiang D., Gao F., Yao H., Ye Q., Wang Y. (2020). The Inhibition of P-Selectin Reduced Severe Acute Lung Injury in Immunocompromised Mice. Oxidative Med. Cell. Longev..

[4] Ning L., Shishi Z., Bo W., Huiqing L. (2023). Targeting immunometabolism against acute lung injury. Clin. Immunol..

[5] Wang Y.-Z., Zhang J., Zhao Y.-L., Li T., Shen T., Li J.-Q., Li W.-Y., Liu H.-G. (2013). Mycology, cultivation, traditional uses, phytochemistry and pharmacology of *Wolfiporia cocos* (Schwein.) Ryvarden et Gilb.: A review. J. Ethnopharmacol..

[6] Liu X., Wang X., Xu X., Zhang X. (2019). Purification, antitumor and anti-inflammation activities of an alkali-soluble and carboxymethyl polysaccharide CMP33 from Poria cocos. Int. J. Biol. Macromol..

[7] Wang J., Wang A., He H., She X., He Y., Li S., Liu L., Luo T., Huang N., Zou K. (2019). Trametenolic acid B protects against cerebral ischemia and reperfusion injury through modulation of microRNA-10a and PI3K/Akt/mTOR signaling pathways. Biomed. Pharmacother..

[8] Liang J., Zhao M., Xie S., Peng D., An M., Chen Y., Li P., Du B. (2022). Effect of steam explosion pretreatment on polysaccharide isolated from *Poria cocos*: Structure and immunostimulatory activity. J. Food Biochem..

[9] Nataraj A., Govindan S., Ramani P., Subbaiah K.A., Sathianarayanan S., Venkidasamy B., Thiruvengadam M., Rebezov M., Shariati M.A., Lorenzo J.M. (2022). Antioxidant, Anti-Tumour, and Anticoagulant Activities of Polysaccharide from Calocybe indica (APK2). Antioxidants.

[10] Wu Y., Li D., Wang H., Wan X. (2022). Protective Effect of Poria Cocos Polysaccharides on Fecal Peritonitis-Induced Sepsis in Mice through Inhibition of Oxidative Stress, Inflammation, Apoptosis, and Reduction of Treg Cells. Front. Microbiol..

[11] Mousa W.K., Chehadeh F., Husband S. (2022). Microbial dysbiosis in the gut drives systemic autoimmune diseases. Front. Immunol..

[12] Mjösberg J., Rao A. (2018). Lung inflammation originating in the gut. Science.

[13] Wang Z., Xie J., Yang Y., Zhang F., Wang S., Wu T., Shen M., Xie M. (2017). Sulfated *Cyclocarya paliurus* polysaccharides markedly attenuates inflammation and oxidative damage in lipopolysaccharide-treated macrophage cells and mice. Sci. Rep..

[14] Parkin J., Cohen B. (2001). An overview of the immune system. Lancet.

[15] González-Martín M., Corbera J.A., Suárez-Bonnet A., Tejedor-Junco M.T. (2020). Virulence factors in coagulase-positive staphylococci of veterinary interest other than *Staphylococcus aureus*. Veter-Q..

[16] Stopińska K., Radziwoń-Zaleska M., Domitrz I. (2021). The Microbiota-Gut-Brain Axis as a Key to Neuropsychiatric Disorders: A Mini Review. J. Clin. Med..

[17] Lukasova M., Malaval C., Gille A., Kero J., Offermanns S. (2011). Nicotinic acid inhibits progression of atherosclerosis in mice through its receptor GPR109A expressed by immune cells. J. Clin. Investig..

[18] Zhang T., Li Q., Cheng L., Buch H., Zhang F. (2019). *Akkermansia muciniphila* is a promising probiotic. Microb. Biotechnol..

[19] Everard A., Belzer C., Geurts L., Ouwerkerk J.P., Druart C., Bindels L.B., Guiot Y., Derrien M., Muccioli G.G., Cani P.D. (2013). Cross-talk between *Akkermansia muciniphila* and intestinal epithelium controls diet-induced obesity. Proc. Natl. Acad. Sci. USA.

[20] Yang B., Li M., Wang S., Ross R.P., Stanton C., Zhao J., Zhang H., Chen W. (2021). *Lactobacillus ruminis* Alleviates DSS-Induced Colitis by Inflammatory Cytokines and Gut Microbiota Modulation. Foods.

[21] Koh A., De Vadder F., Kovatcheva-Datchary P., Bäckhed F. (2016). From Dietary Fiber to Host Physiology: Short-Chain Fatty Acids as Key Bacterial Metabolites. Cell.

[22] Shu-Guang H.E. (2012). Distribution and drug resistance of 216 strains of *Chryseobacterium* causing nosocomial infection. Chin. J. Nosocomiology.

[23] Cong S., Wang L., Meng Y., Cai X., Zhang C., Gu Y., Ma X., Luo L. (2023). *Saussurea involucrata* oral liquid regulates gut microbiota and serum metabolism during alleviation of collagen-induced arthritis in rats. Phytother. Res..

[24] Zong S., Ye H., Ye Z., He Y., Zhang X., Ye M. (2022). Polysaccharides from *Lachnum* sp. Inhibited colitis-associated colon tumorigenesis in mice by modulating fecal microbiota and metabolites. Int. Immunopharmacol..

[25] Liu Q., Tian X., Maruyama D., Arjomandi M., Prakash A. (2021). Lung immune tone via gut-lung axis: Gut-derived LPS and short-chain fatty acids’ immunometabolic regulation of lung IL-1β, FFAR2, and FFAR3 expression. Am. J. Physiol.-Lung Cell. Mol. Physiol..

[26] Ebeid T.A., Al-Homidan I.H. (2022). Organic acids and their potential role for modulating the gastrointestinal tract, antioxidative status, immune response, and performance in poultry. World’s Poult. Sci. J..

[27] Li L., Fu W.W., Wu R.T., Song Y.H., Wu W.Y., Yin S.H., Li W.J., Xie M.Y. (2020). Protective effect of Ganoderma atrum polysaccharides in acute lung injury rats and its metabolomics. Int. J. Biol. Macromol..

[28] Murakami Y., Hoshi M., Hara A., Takemura M., Arioka Y., Yamamoto Y., Matsunami H., Funato T., Seishima M., Saito K. (2012). Inhibition of increased indoleamine 2,3-dioxygenase activity attenuates Toxoplasma gondii replication in the lung during acute infection. Cytokine.

[29] Swolana D., Wojtyczka R.D. (2022). Activity of Silver Nanoparticles against *Staphylococcus* spp.. Int. J. Mol. Sci..

[30] Du L., Zhang J., Zhang X., Li C., Wang Q., Meng G., Kan X., Zhang J., Jia Y. (2022). Oxypeucedanin relieves LPS-induced acute lung injury by inhibiting the inflammation and maintaining the integrity of the lung air-blood barrier. Aging.

[31] Fujishima S., Aikawa N. (1995). Neutrophil-mediated tissue injury and its modulation. Intensive Care Med..

[32] Meng Yun L.B. (2021). The Correlation Between the Levels of Blood Uric Acid IL-2 IL-6 and TNF-αand Scores of Depressive Symptoms and Somatiization Sympton in Patiens with Depression. Hebei Med..

[33] Wang H., Zhou C., Huang J., Kuai X., Shao X. (2020). The potential therapeutic role of *Lactobacillus reuteri* for treatment of inflammatory bowel disease. Am. J. Transl. Res..

