# Supplementary material for: Research on Pachymaran to Ameliorate CsA-Induced Immunosuppressive Lung Injury by Regulating Microflora Metabolism
Source: Microorganisms. 2023 Sep 7;11(9):2249. doi: 10.3390/microorganisms11092249 (PMC10537689; doi:10.3390/microorganisms11092249)
Supplement: Supplementary file 1 [file microorganisms-11-02249-s001.zip › microorganisms-2569693-supplementary.pdf]

Supplementary Materials

Figure S1. PCP regulates the microecological diversity and composition of lung tissue. Note: Z: control group (n=5); M: model group (n=5); P/PCP: PCP group (n=5). (**A**) PLS-DA analysis (lung microecology) (**B-C**) LEfSe analysis (lung microecology), LDA>3.

Figure S2. PCP regulates the diversity and composition of the gut microbiota. Note: Z: control group (n=5); M: model group (n=5); P/PCP: PCP group (n=5). (**A**) Species distribution map of microbial community at gate level (lung microecology) (**B-D**) LEfSe analysis (gut microecology), LDA>4.

Figure S3. PCP regulates the diversity and composition of the gut microbiota. Note: Z: control group (n=5); M: model group (n=5); P/PCP: PCP group (n=5). (**A**) PLS-DA analysis (gut microecology) (**B**) Species distribution map of microbial community at phylum level (gut microecology) (**C-D**) Wilcoxon test (p<0.05) for relative abundance analysis of gut flora.

Figure S4. Differentially expressed metabolites (DEM). Note: Control group (n=5); Model group (n=5); PCP: PCP group (n=5). The LC-MS Identified 178 significantly altered DEM, p<0.05.

Figure S5. 20 species of the most relevant DEM. (**A**)Model VS control: 20 different metabolites (Correlation analysis uses the Pearson correlation coefficient to measure the degree of linear correlation between two metabolites. Red indicates a positive correlation and blue indicates a negative correlation. A larger dot indicates a higher correlation coefficient between the two variables.) (**B**) PCP VS Model: 20 different metabolites.
