# Supplementary figures and images for: Research on Pachymaran to Ameliorate CsA-Induced Immunosuppressive Lung Injury by Regulating Microflora Metabolism
Source: Microorganisms. 2023 Sep 7;11(9):2249. doi: 10.3390/microorganisms11092249 (PMC10537689; doi:10.3390/microorganisms11092249)

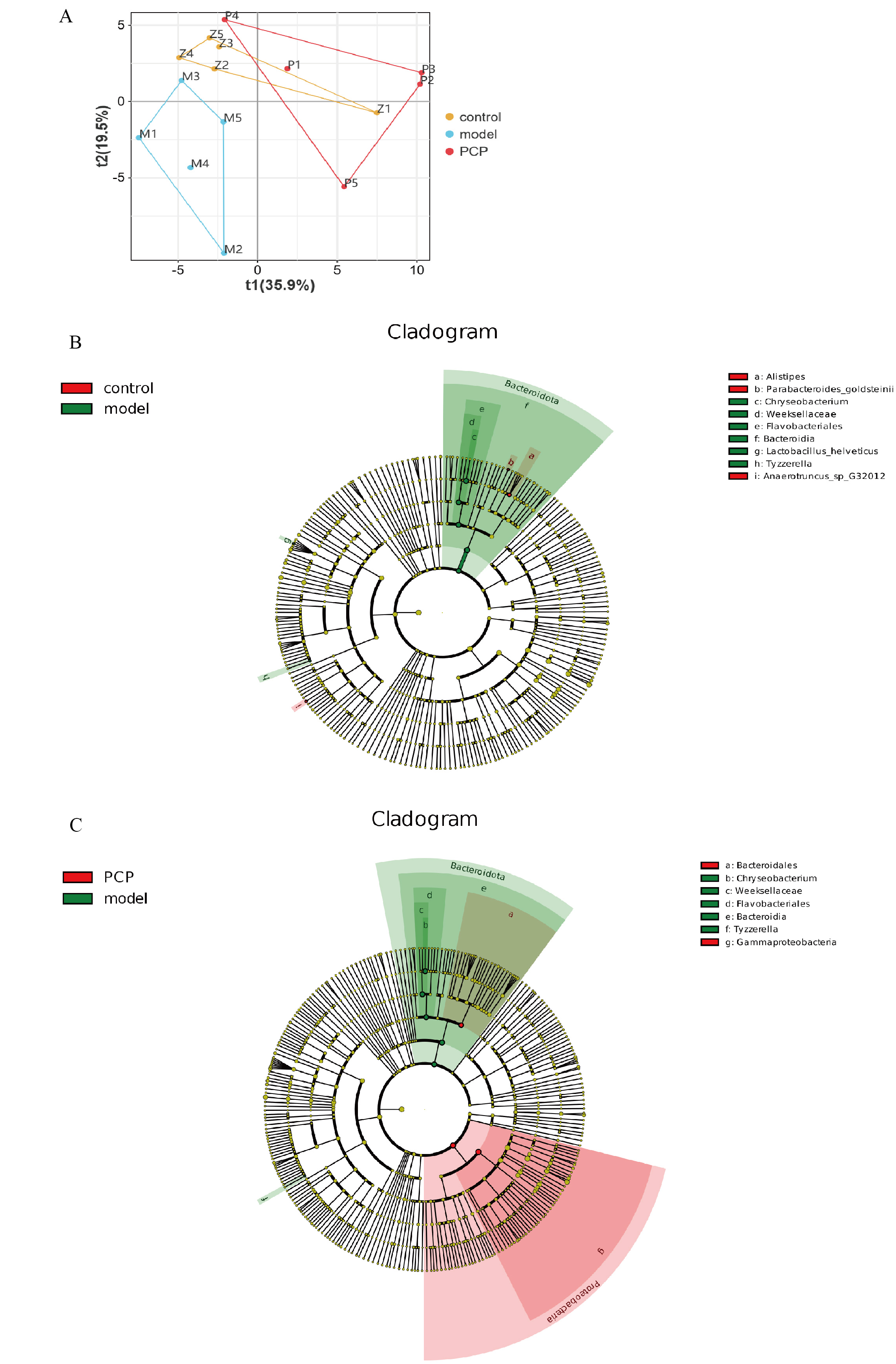

Supplement: Supplementary file 1 [file microorganisms-11-02249-s001.zip › Supplementary.Figure-S1.jpg]

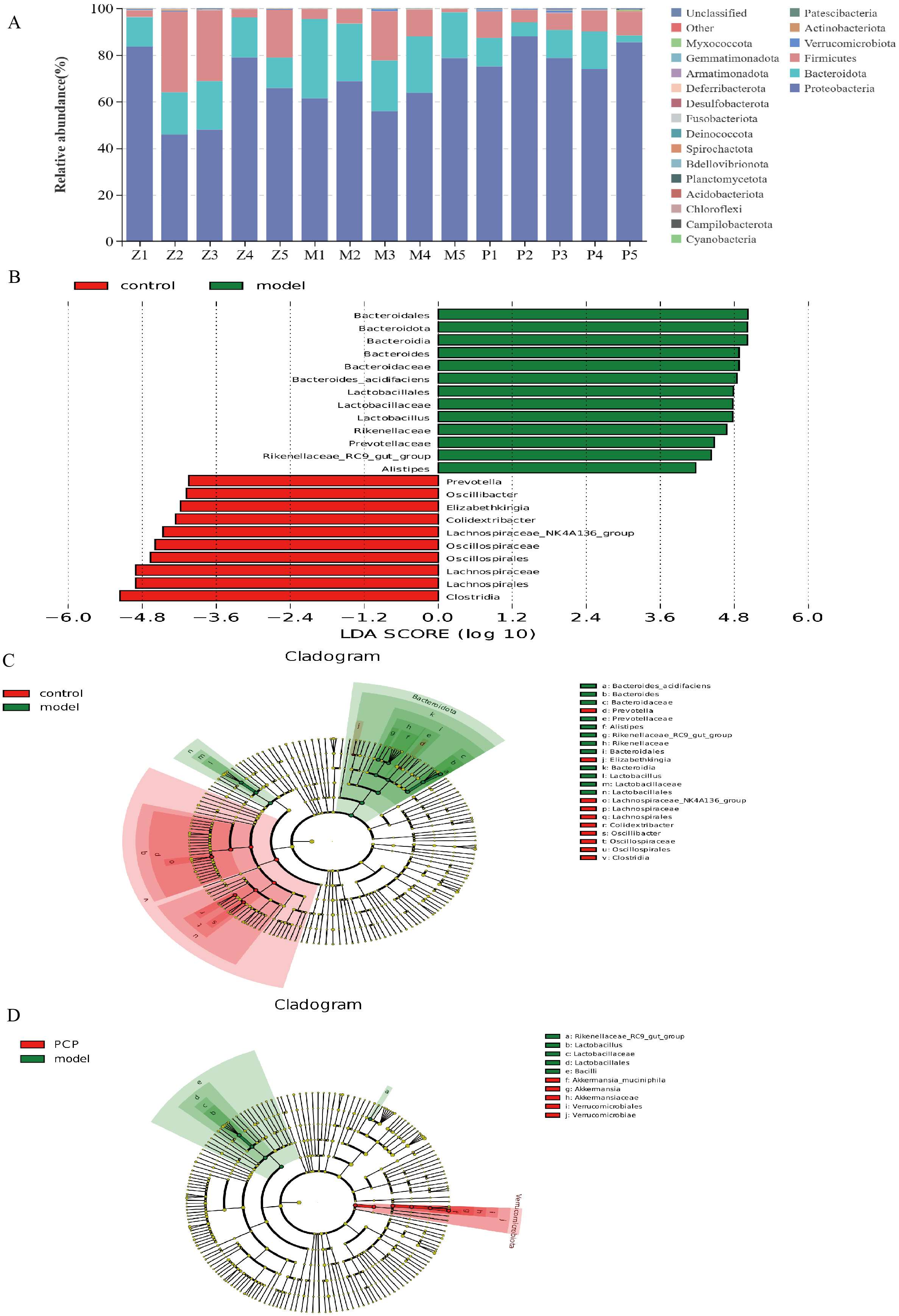

Supplement: Supplementary file 1 [file microorganisms-11-02249-s001.zip › Supplementary.Figure-S2.jpg]

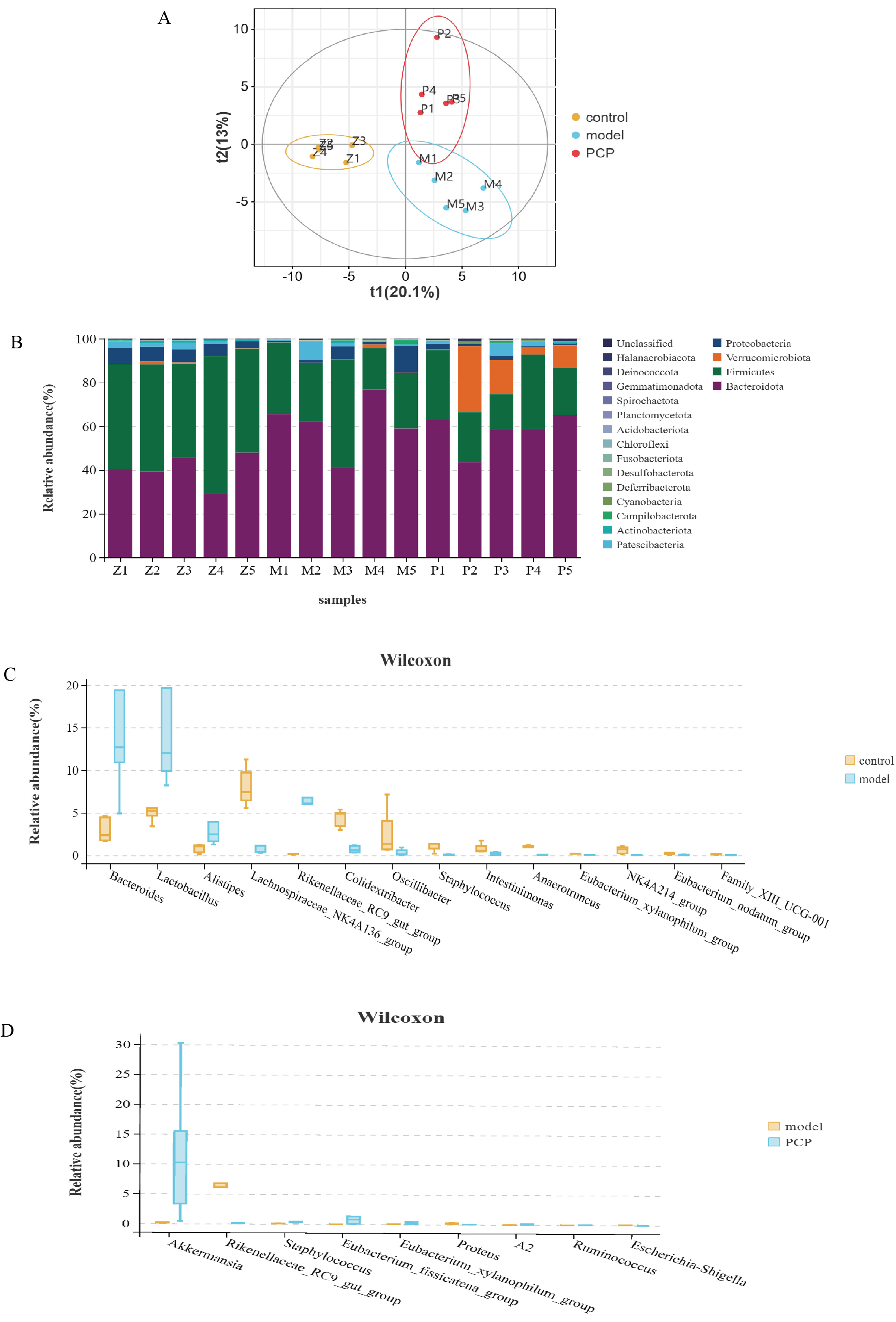

Supplement: Supplementary file 1 [file microorganisms-11-02249-s001.zip › Supplementary.Figure-S3.jpg]

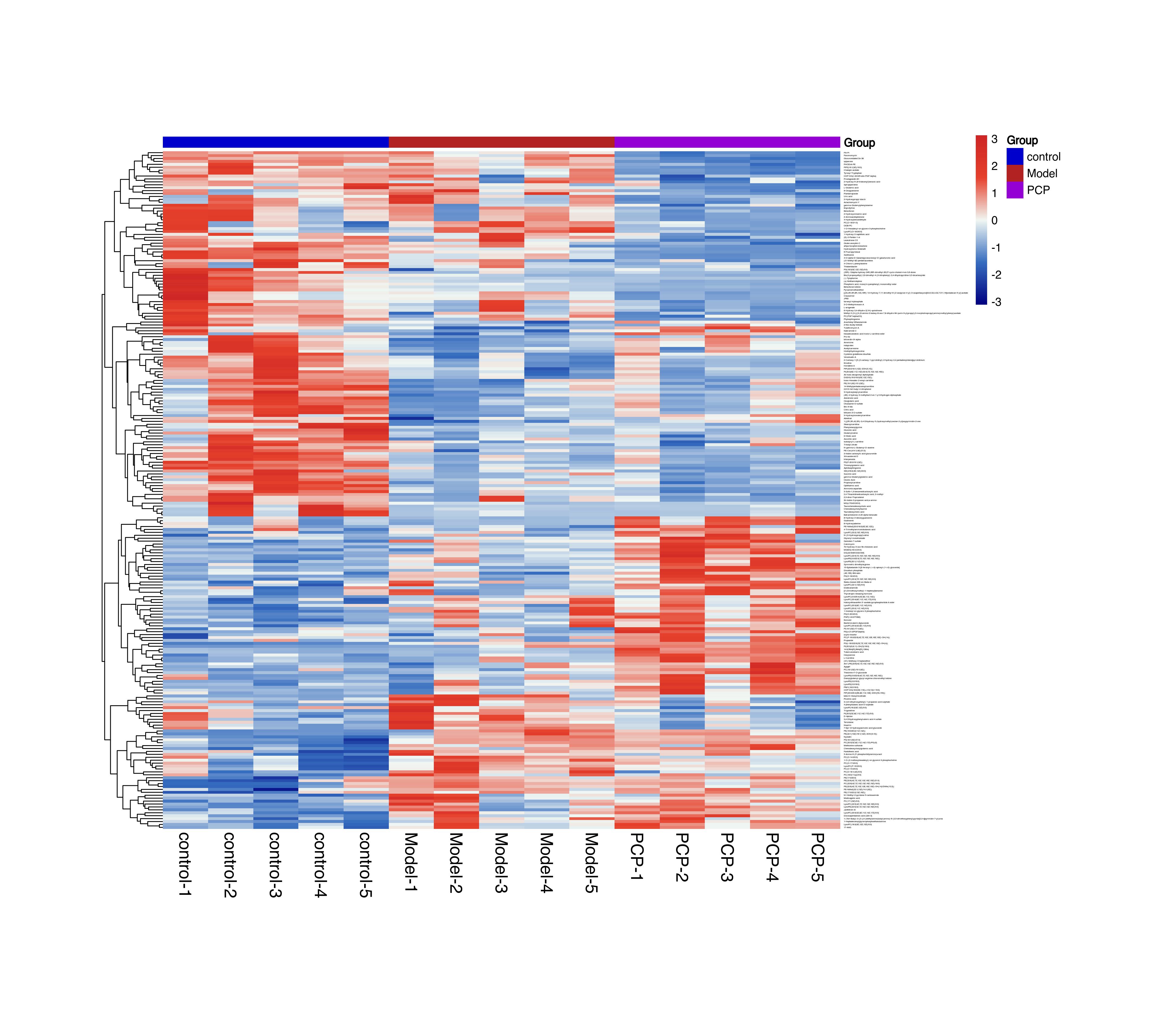

Supplement: Supplementary file 1 [file microorganisms-11-02249-s001.zip › Supplementary.Figure-S4.jpg]

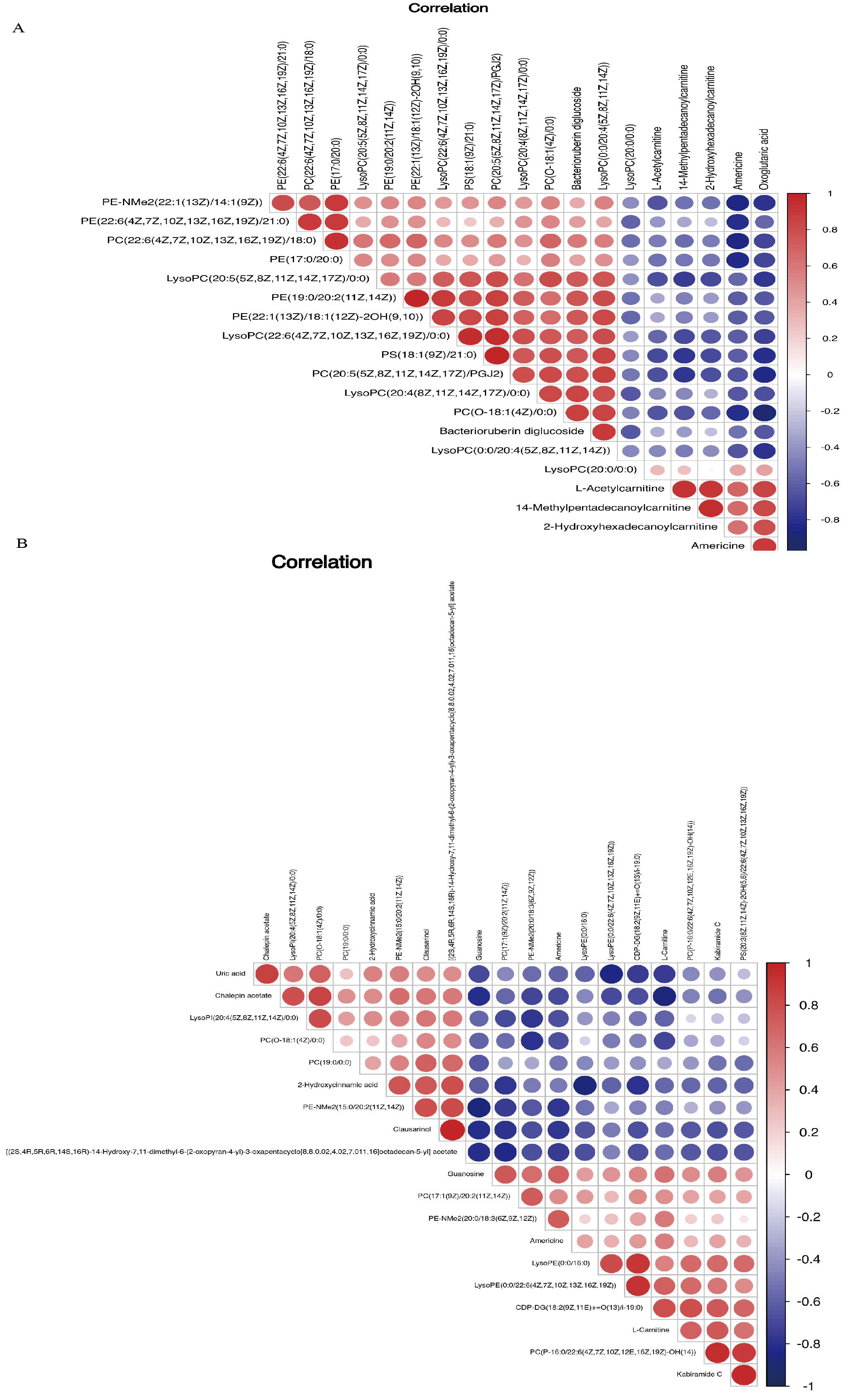

Supplement: Supplementary file 1 [file microorganisms-11-02249-s001.zip › Supplementary.Figure-S5.jpg]
